# Toward the Role of Language Teacher Confirmation and Stroke in EFL/ESL Students’ Motivation and Academic Engagement: A Theoretical Review

**DOI:** 10.3389/fpsyg.2021.723432

**Published:** 2021-07-19

**Authors:** Yun Gao

**Affiliations:** School of Foreign Languages, Xinyang College, Xinyang, China

**Keywords:** theoretical review, confirmation, stroke, language teacher, motivation, academic engagement, EFL/ESL students

## Abstract

Following the recent special issue in the journal of Frontiers in Psychology, named “*The Role of Teacher Interpersonal Variables in Students’ Academic Engagement, Success, and Motivation*,” this review is carried out to describe two prime instances of teacher interpersonal behaviors, namely teacher confirmation and stroke, their underlying frameworks, and contributions to desirable student-related outcomes. In light of rhetorical-relational goal theory and the school of positive psychology, it is stipulated that language teacher confirmation and stroke are facilitators of EFL/ESL students’ level of motivation and academic engagement. Providing empirical evidence, the argument regarding the pivotal role of language teacher confirmation and stroke in EFL/ESL contexts was proved. Reviewing the available literature on the aforementioned variables, some pedagogical implications were suggested for teacher trainers, educational supervisors, and pre- and in-service language teachers. Finally, the limitations and drawbacks of the reviewed studies were identified and some avenues for further research were recommended, accordingly.

## Introduction

The impact of teacher interpersonal variables on students’ academic engagement, success, and motivation is the focus of a recent special issue in the journal of “*Frontiers in Psychology.*” In an effort to respond to this call, the present theoretical review aims to delineate two important interpersonal behaviors of teachers, namely teacher confirmation and stroke, their underlying theoretical frameworks, as well as their capability in predicting EFL/ESL students’ motivation and academic engagement in instructional-learning environments. As put forward by Pekrun and Schutz (2007), desirable student-related outcomes can be attained in an educational atmosphere where in students enjoy their learning experiences. Among different factors providing pleasant learning experiences for students in educational contexts, positive teacher-student interpersonal relationship is of great significance (Gałajda et al., 2016; Wendt and Courduff, 2018; Sun et al., 2019). To put it simply, the teacher-student relationship is one of the fundamental building blocks for an effective instructional-learning environment. This is mainly due to the fact that both teachers and students are equally responsible for the effective implementation of the learning and teaching processes; hence, they have to collaborate to create a favorable learning condition (Gabryś-Barker and Gałajda, 2016). Given the importance of teacher-student interpersonal relationship, an increasing attention has been paid to its essence and quality (e.g., Yu and Zhu, 2011; Zhang, 2011; Zhang and Sapp, 2013; Zhu, 2013; Pishghadam and Khajavy, 2014; Wei et al., 2015; Henry and Thorsen, 2018; Wendt and Courduff, 2018; Nayernia et al., 2020; Derakhshan, 2021; Pishghadam et al., 2021).

In any interaction, teachers and students may affect each other either negatively or positively (Zhong, 2013; Brinkworth et al., 2018; Pishghadam et al., 2019; Luo et al., 2020). While a negative teacher-student relationship may result in several adverse consequences (e.g., anxiety, burnout, depression, etc.), a positive teacher-student connection can have a desirable impact on students’ motivation and academic engagement (Roorda et al., 2011; Van Uden et al., 2014; Martin and Collie, 2019; Derakhshan, 2021). Employing appropriate interpersonal behaviors, teachers can develop such a positive relationship with their students. Interpersonal behaviors are verbal, para-verbal, and non-verbal in nature (Babonea and Munteanu, 2012; Dobrescu and Lupu, 2015; Tranca and Neagoe, 2018). Verbal interpersonal communication refers to “a two-way exchange that involves both talking and listening” (Babonea and Munteanu, 2012, p. 2). Such interpersonal communication is crucial in forming bonds and developing relationships between teachers and their students. Verbal interpersonal behaviors enable teachers to draw students’ attention during their instruction. In the case of para-verbal interpersonal communication, “the message is not transmitted through words, but could not get to the listeners without speaking” (Babonea and Munteanu, 2012, p. 2). As put forward by Tranca and Neagoe (2018), Para-verbal interpersonal communication is directly related to the “intonation,” “the volume of voice,” “the intensity of voice,” “the tone of voice,” and “the speech rate.” Finally, non-verbal interpersonal communication “uses as tools physical appearance, facial expression, and gesture, which give nuances to the message” (Babonea and Munteanu, 2012, p. 3). Non-verbal interpersonal communication behaviors are tied with “posture,” “hand gestures,” “body movements,” “facial expressions,” and “eye contact” (Tranca and Neagoe, 2018).

Teacher care, credibility, clarity, confirmation, stroke, humor, immediacy, and praise are examples of verbal, para-verbal, and non-verbal interpersonal behaviors. Among them, this review mainly focuses on confirmation and stroke as the prime instances of teacher positive interpersonal behaviors. The following sections explain these two interpersonal behaviors, their underpinning frameworks, as well as two student-related variables, namely motivation and academic engagement, empirically proved to be predicted by the aforementioned interpersonal behaviors.

### Teacher Confirmation

For more than four decades, the term “confirmation” has emerged in theological, philosophical, and communication literature. Several scholars have highlighted the pivotal role of confirmation in teacher-student interpersonal relationships (e.g., Goodboy and Myers, 2008; Schrodt and Finn, 2011; Goldman et al., 2014; Goldman and Goodboy, 2014; Hsu and Huang, 2017; Geier, 2020). Ellis (2000) referred to teacher confirmation as “the transactional process by which teachers talk and interact with students that make them feel they are valuable and significant individuals” (p. 265). The concept of teacher confirmation dates back to the “Broaden-and-Build Theory” (Fredrickson, 2001) and “Emotional Response Theory” (Mottet and Beebe, 2006).

Emotional Response Theory was designed to describe the intricacies of the interaction between “teacher communication behaviors” and “students’ behavioral and emotional responses” (Mottet et al., 2006, p. 259). As put forward by Mottet et al. (2006), relationally-oriented teaching behaviors such as confirmation have an enormous effect on students’ feelings of arousal, enjoyment, and dominance, which in turn encourages students to participate in approach behaviors toward learning. Fredrickson’s (2001) broaden-and-build theory also suggests that students’ cognitive abilities can be dramatically enhanced by experiencing positive feelings. However, the degree of teacher confirmation appears to have a significant impact on this association (Schrodt and Finn, 2011). To put it differently, students’ conduct and attitudes toward instruction and instructional-learning context can be improved by receiving confirmation from others, illustrating the considerable association between teacher confirmation behaviors and student-related outcomes (Goldman et al., 2018).

Ellis (2000) characterized teacher confirmation around four major dimensions, namely “responding to students’ questions and/or comments,” “demonstrating interest in the student’s learning process,” “employing an interactive teaching style in the classroom,” and “absence of general disconfirmation” (p. 266). The first dimension relates to how much time teachers devote to answering students’ questions effectively, and to what extent teachers take time to listen to students’ questions carefully. The second dimension is concerned with teachers’ interests in students’ learning process, or whether teachers are sufficiently enthusiastic about their students’ study process. The third dimension refers to what extent teachers employ interactive teaching methods to assist students in understanding course content. Employing an interactive teaching style, teachers can modify their teaching practices based on their students’ needs and interests. Finally, the fourth dimension of teacher confirmation is tied with not exhibiting any disconfirming actions toward students, which can be deemed as a kind of confirmation (Ellis, 2004). Previous research has revealed that teacher confirmation behavior is associated with EFL/ESL student-related variables such as increased achievement (Goodboy and Myers, 2008; Hsu, 2012; Goldman et al., 2014; Santana, 2017), motivation (Ellis, 2004; Shen and Croucher, 2018; Croucher et al., 2021), and academic engagement (Campbell et al., 2009; LaBelle and Johnson, 2020).

### Teacher Stroke

The notion of stroke is conceptualized as a unit of human recognition that may be used to meet an individual’s desire for recognition (Berne, 1988). It may be traced back to Berne’s (1988) transactional analysis (TA). As put forward by Stewart and Joines (1987), TA is “a theory of personality and a systematic psychotherapy for personal growth and personal change” (p. 4). Newell and Jeffery (2002) categorized TA into six components of “strokes,” “time structures,” “ego states,” “life positions,” “life scenario,” and “transactions.” Regarding the significance of stroke, the first component of TA, Berne et al. (2011) stated that stroke is a crucial element in improving our life quality. Individuals employ a variety of strategies to give and accept a stroke. In this regard, Stewart and Joines (1987) have divided stroke into three dichotomous categories: verbal/non-verbal, positive/negative, and conditional/unconditional. Verbal strokes include the exchange of speech, ranging from saying a single word to maintaining a lengthy conversation. Non-verbal strokes, on the other hand, cover a wide range of non-verbal actions such as smiling and nodding. Negative strokes cause dissatisfaction, whereas positive strokes result in happiness, enjoyment, and satisfaction. In terms of conditional and unconditional strokes, Berne (1988) expounded that “unconditional strokes are related to what you are, while conditional strokes are about what you do” (Pishghadam et al., 2019, p. 286).

The concept of stroke is commonly used in educational psychology to refer to “teacher feedback” and “teacher praise” (Amini et al., 2019). In Instructional-learning contexts, instructors can stroke students in a number of ways, including “calling students by their names,” “allowing them to express themselves,” and “offering adequate feedbacks” (Amini et al., 2019, p. 28). According to Hattie and Timperley (2007), a stroke-rich instructional setting motivates EFL/ESL students to perform better. Similarly, Rajabnejad et al. (2017) hold that teachers’ stroke increases students’ tendency to repeat desirable behaviors that are essential for their academic success. Previous research proved that teacher strokes enable them to promote EFL/ESL students’ willingness to attend classes (Pishghadam et al., 2021), intelligence, care, and feedback (Derakhshan et al., 2019), motivation (Akin-Little et al., 2004; Pishghadam and Khajavy, 2014), as well as academic engagement (Van Uden et al., 2014).

### Student Motivation

In a broad sense, motivation deals with individuals’ motive “to make certain decisions,” “to participate in the activity,” and “to persist in pursuing it” (Ushioda, 2008). With regard to language learning, Gardner (1985) defined motivation as the degree of effort a person expends to learn language. Dörnyei (2005) also referred to language motivation as the primary impetus for initiating language learning as well as the reason for continuing the prolonged and tedious process of learning.

Katt and Condly (2009) classified student motivation to learn a language into two main categories of “trait motivation” and “state motivation.” Trait motivation is “a general tendency toward learning,” whereas state motivation is “an attitude toward a particular course” (Katt and Condly, 2009, p. 217). While students’ trait motivation inclines to be stable, their state motivation can be directly or indirectly influenced by their teachers’ interpersonal behaviors (Fallah, 2014). Hence, in interaction with students, teachers should employ positive interpersonal behaviors such as confirmation (Ellis, 2004; Shen and Croucher, 2018; Croucher et al., 2021) and stroke (Akin-Little et al., 2004; Pishghadam and Khajavy, 2014) to enhance EFL/ESL student motivation to learn language.

### Student Academic Engagement

The definition and scope of student engagement vary considerably. That is, almost all scholars conceptualized this concept differently. Lamborn et al. (1992), for instance, defined student engagement as “students’ psychological effort and investment toward learning, understanding, or mastering the skills, crafts, or knowledge that the coursework is intended to promote” (p. 13). Later, Skinner et al. (2009) referred to this concept as “the quality of students’ participation or connection with the educational endeavor and hence with activities, values, people, goals, and place that comprise it” (p. 495). Despite the fact that the scope and conceptualization of student engagement are extremely different, scholars have agreed upon the multidimensionality of this concept. They believe that student academic engagement covers a number of factors that work together to display students’ positive feelings toward the learning process (Fredricks et al., 2004; Christenson et al., 2008; Reeve and Tseng, 2011; Carter et al., 2012; Upadyaya and Salmela-Aro, 2013; Phan, 2014; Lei et al., 2018; Zhao et al., 2020, 2021).

As shown in Table 1, similar to the definition and scope of student engagement, there is debate over the types and number of its components (Appleton et al., 2008; Li and Lerner, 2011). For instance, Schaufeli et al. (2002) divided student engagement into three components of “Vigor,” “Absorption,” and “Dedication,” as opposed to Appleton et al. (2006) who categorized this concept across four dimensions of “Academic,” “Behavioral,” “Psychological,” and “Cognitive.”

**TABLE 1:** Variations in categorizing the components of student engagement.

**Authors**	**Components of student engagement**
Audas and Willms (2002)	• Behavioral: “Engaging in class activities” • Psychological: Encompasses aspects such as “sense of belonging, relationships with teachers and peers, and valuing school outcomes”
Appleton et al. (2006)	• Academic: Reflected by indicators such as “time on task, “homework completion, and credit earned toward graduation” • Behavioral: “Attendance, classroom participation, suspensions, and participation in extracurricular activities” • Psychological: “Having sense of belonging or identification,” and “relationships with peers and teachers” • Cognitive: “Self-regulated learning, valuing of learning, autonomy, and personal aims”
Finn (1989)	• Participation: “Engaging in classrooms activities/tasks” • Identification: “Feeling of belonging in school and valuing learning-related outcomes”
Fredricks et al. (2004)	• Behavioral: “Students’ participation in academic and extracurricular activities” • Emotional: “Students’ positive and negative reaction to peers, teachers, and schools” • Cognitive: “Students’ thoughtfulness and willingness to master difficult skills”
Jimerson et al. (2003)	• Affective: Feelings about “the educational institutions, teachers, and peers” • Behavioral: Includes students’ “observable performance and action” • Cognitive: Encompasses students’ “beliefs and perceptions related to self, academic institutions, teachers, and peers”
Reeve and Tseng (2011)	• Behavioral: “Students’ engagement in learning activities such as effort, persistence, and attention” • Emotional: “Students’ presence of enthusiasm and interest, lack of anger, boredom, and anxiety” • Cognitive: “Student’s use of active self-regulation and sophisticated learning strategies” • Agentic: “Students’ constructive contribution toward the flow of the instruction he receives”
Schaufeli et al. (2002)	• Vigor: “Persistence, resilience, and effort in the face of difficulties” • Absorption: “Engrossment in tasks and activities of learning” • Dedication: “Inspiration, pride, and enthusiasm in academic learning”
Willms (2003)	• Behavioral: “Engaging in academic and non-academic school-related activities” • Psychological: “Sense of attachment or belonging to school, and valuing school outcomes”

As put forward by many scholars (e.g., Upadyaya and Salmela-Aro, 2013; Salmela-Aro and Upadyaya, 2014; Alrashidi et al., 2016; Hiver et al., 2021), among different models of student engagement, the models of Schaufeli et al. (2002) and Fredricks et al. (2004) have received more attention in realizing and explaining the complex nature of the student engagement construct.

Previous empirical studies have examined the construct of student academic engagement from various angles, namely as a means of improving learning outcomes (Parsons et al., 2014; Wonglorsaichon et al., 2014; Virtanen et al., 2015), a way of minimizing school dropout (Janosz et al., 2008), and an indicator of student academic motivation (Akpan and Umobong, 2013; Wu, 2019; Ghanizadeh et al., 2020; Ghelichli et al., 2020; Muñoz-Restrepo et al., 2020).

### The Role of Teacher Confirmation and Stroke in EFL/ESL Students’ Motivation and Academic Engagement

Compared to other positive interpersonal behaviors of language teachers, confirmation and stroke have been the focus of less empirical research. However, the available literature on language teacher confirmation and stroke reported valuable findings regarding the impact of these interpersonal behaviors on EFL/ESL student-related variables, notably student motivation and academic engagement.

With regard to EFL/ESL student motivation, Ellis (2009) found that language teacher confirmation directly affects student motivation to learn the language. Similarly, Goodboy and Myers (2008) reported a positive association between language teachers’ confirmation behaviors and students’ state and trait motivation. As far as teacher stroke is concerned, Amini et al. (2019) found a statistically significant relationship between teacher stroke and EFL students’ increased motivation. By the same token, Pishghadam and Khajavy (2014) uncovered that teacher stroke can remarkably predict EFL students’ intrinsic and extrinsic motivation to learn the language. In the same vein, Croucher et al. (2021) demonstrated that ESL students’ level of academic motivation can be dramatically enhanced by teacher confirmation behaviors.

Concerning EFL/ESL student academic engagement, Campbell et al. (2009) showed that language teacher confirmation can lead to higher student motivation in EFL/ESL classes. Similarly, Waldbuesser (2019) found that a positive relationship exists between language teacher confirmation and student academic engagement. When it comes to teacher stroke, Van Uden et al. (2014) reported that language teacher stroke is directly associated with students’ higher engagement. In this regard, Baños et al. (2019) also demonstrated that language teachers can increase students’ academic engagement in a stroke-rich instructional context.

The empirical evidence on the role of teacher confirmation and stroke in enhancing EFL/ESL students’ motivation and academic engagement can be justified by the “rhetorical-relational goal theory” (Mottet et al., 2006). This theory is grounded on the following key premises (Myers et al., 2018, p. 2):

• Teachers have some relational and rhetorical goals.
• Students have some relational and academic needs and wants.
• Effective teaching is the consequence of defining proper rhetorical and relational goals and employing effective interpersonal behaviors to obtain those goals.
• Students who feel more satisfied in the instructional-learning environment and whose relational and academic expectations, needs, and wants are addressed, are more inclined to engage in the learning process.
• Grade levels and instructional contexts influence teachers’ relational and rhetorical goals.
• Students’ relational and academic needs and desires differ across different phases of development.

According to the aforementioned assumptions of rhetorical-relational goal theory, it can be reasonably inferred that teachers can fulfill students’ academic needs and wants through employing positive interpersonal behaviors, which in turn result in some favorable consequences such as increased motivation and academic engagement (Houser and Hosek, 2018).

The predictability of EFL/ESL students’ motivation and academic engagement through teacher confirmation and stroke have also something to do with “positive psychology” (Seligman, 2018; Dewaele et al., 2019; Li and Xu, 2019). Positive psychology is founded on three core elements, including “positive experiences,” “positive individual traits,” and “positive institutions” (Seligman and Csikszentmihalyi, 2000, p. 9). This school of thought posits that positive interaction between teachers and students provides an appropriate instructional-learning environment in which students enjoy their learning experiences (MacIntyre et al., 2016; Budzińska and Majchrzak, 2021). As put forward by Seligman (2011), enjoying the learning process is a key factor in promoting student-related outcomes such as academic engagement and motivation.

## Discussion

In this theoretical review, two prime instances of positive interpersonal behaviors (i.e., confirmation and stroke), their underlying frameworks (i.e., broaden-and-build theory, emotional response theory, and transactional analysis), and their associations with two favorable student-related outcomes (i.e., motivation and academic engagement) were explained. Furthermore, two critical theoretical frameworks (i.e., rhetorical-relational goal theory and positive psychology) that support the connections among the aforementioned variables were fully described.

According to what was theoretically reviewed, it appears that this research area has some pedagogical implications for teacher trainers, educational supervisors, and pre- and in-service language teachers. For instance, teacher trainers should alter teachers’ attitudes and perceptions toward their educational responsibilities. To put it simply, they should make teachers aware that their responsibilities in instructional-learning environments are beyond the transmission of content and pedagogical knowledge. That is, teachers must be equipped with the knowledge that the interpersonal behaviors they employ in interaction with their students (e.g., confirmation and stroke) are as important as their knowledge and instructional skills.

Moreover, educational supervisors who are responsible for observing teachers and evaluating their academic effectiveness can take some advantage of research evidence in the area of the positive teacher-student relationships. Given the pivotal role of teachers’ interpersonal behaviors in the adequacy of instruction and learning, besides teacher competence and instructional skills, supervisors should also take interpersonal behaviors into account as other essential dimensions of teacher success. Last but by no means the least, both pre- and in-service language teachers should enhance their effectiveness by pursuing recent studies on effective interpersonal behaviors of teachers, attending different related conferences and workshops, and evaluating their own interpersonal behaviors. In light of new information, language teachers should make necessary changes in the interpersonal behaviors they employ in interactions with their EFL/ESL students.

Finally, a number of important limitations need to be noted regarding the available literature on teacher confirmation and stroke. To start with, compared to other positive interpersonal behaviors, teacher confirmation and stroke have received less attention (e.g., Sidelinger and Booth-Butterfield, 2010; Hsu, 2012); hence, more empirical studies should be conducted on these two interpersonal behaviors. Next, previous research proved that teacher confirmation and stroke play a pivotal role in improving student motivation and academic engagement (Van Uden et al., 2014; LaBelle and Johnson, 2020; Croucher et al., 2021); it would be interesting to examine whether other student-related variables (e.g., learning achievement, willingness to communicate, satisfaction, etc.) can be positively predicted by teacher confirmation and stroke.

Moreover, almost all previous studies employed a quantitative method to examine the interplay of language teacher confirmation and stroke with respect to student motivation and academic engagement. What is now needed is to examine the inter-relationships of these variables through qualitative and mixed-method approaches that offer a more detailed understanding of the subject under inquiry (Patton, 2015; Ary et al., 2018). Furthermore, the questionnaire was the sole instrument employed in these studies. For the sake of triangulation, future studies can employ other data collection instruments such as interviews, observation schemes, and diary writing. Additionally, the majority of the existing literature has been carried out in EFL contexts. As such, future research needs to be conducted to uncover the extent to which the association among the aforementioned variables is present in ESL instructional-learning contexts.

A further limitation of the available literature deals with the measurement of teacher interpersonal variables. That is, the majority of studies merely used observer-report scales to measure teacher stroke and confirmation. Hence, the attitudes and perceptions of teachers toward their interpersonal behaviors were neglected. To fill this gap, further empirical studies are advised to use both observer-report scales and self-report scales to measure these two interpersonal behaviors of teachers. Last but not least, the moderating effects of situational variables such as gender (Campbell et al., 2009) and culture (Shen and Croucher, 2018; Croucher et al., 2021) have been the focus of less research. In this regard, more empirical research is highly required to examine the impact of different situational variables.

## Author Contributions

The author confirms being the sole contributor of this work and has approved it for publication.

## Conflict of Interest

The author declares that the research was conducted in the absence of any commercial or financial relationships that could be construed as a potential conflict of interest.
